# Accuracy of Breast Ultrasonography and Mammography in Comparison with Postoperative Histopathology in Breast Cancer Patients after Neoadjuvant Chemotherapy

**DOI:** 10.3390/diagnostics13172811

**Published:** 2023-08-30

**Authors:** Gilda Schmidt, Sebastian Findeklee, Gerda del Sol Martinez, Mihai-Teodor Georgescu, Christoph Gerlinger, Sogand Nemat, Gilbert Georg Klamminger, Meletios P. Nigdelis, Erich-Franz Solomayer, Bashar Haj Hamoud

**Affiliations:** 1 Department for Gynecology, Obstetrics and Reproductive Medicine, Saarland University Hospital, 66421 Homburg, Germany; gilda.schmidt@uks.eu (G.S.); bashar.hajhamoud@uks.eu (B.H.H.); 2 “Prof. Dr. Al. Trestioreanu” Oncology Discipline, “Carol Davila” University of Medicine and Pharmacy, 020021 Bucharest, Romania; 3 “Prof. Dr. Al. Trestioreanu” Oncology Institute, 022328 Bucharest, Romania; 4 Clinic for Diagnostic and Interventional Radiology, Medical Faculty, Saarland University, 66421 Homburg, Germany; 5 Unit of Reproductive Endocrinology, 1st Department of Obstetrics and Gynecology, Papageorgiou General Hospital, Aristotle University of Thessaloniki, 564 03 Thessaloniki, Greece

**Keywords:** breast cancer, neoadjuvant chemotherapy, breast ultrasonography, mammography, pathological complete response

## Abstract

Introduction: Nowadays chemotherapy in breast cancer patients is optionally applied neoadjuvant, which allows for testing of tumor response to the chemotherapeutical treatment in vivo, as well as allowing a greater number of patients to benefit from a subsequent breast-conserving surgery. Material and methods: We compared breast ultrasonography, mammography, and clinical examination (palpation) results with postoperative histopathological findings after neoadjuvant chemotherapy, aiming to determine the most accurate prediction of complete remission and tumor-free resection margins. To this end, clinical and imaging data of 184 patients (193 tumors) with confirmed diagnosis of breast cancer and neoadjuvant therapy were analyzed. Results: After chemotherapy, tumors could be assessed by palpation in 91.7%, by sonography in 99.5%, and by mammography in 84.5% (chi-square *p* < 0.0001) of cases. Although mammography proved more accurate in estimating the exact neoadjuvant tumor size than breast sonography in total numbers (136/163 (83.44%) vs. 142/192 (73.96%), n.s.), 29 tumors could be assessed solely by means of breast sonography. A sonographic measurement was feasible in 192 cases (99.48%) post-chemotherapy and in all cases prior to chemotherapy. Conclusions: We determined a superiority of mammography and breast sonography over clinical palpation in predicting neoadjuvant tumor size. However, neither examination method can predict either pCR or tumor margins with high confidence.

## 1. Introduction

Breast cancer is the most common malignant disease in women [1,2,3]. The worldwide incidence of 2.26 million cases in 2020, according to the global cancer burden, vividly demonstrates the extent of the disease and its global impact. Based on these findings, the WHO recently formed the Global Breast Cancer Initiative to improve overall survival rates, which even today vary dramatically by region [4,5]. To date, the broad spectrum of potential treatment options includes surgical tumor removal, radiation therapy, hormonal therapy, systemic chemotherapy, and targeted therapy. Surgical therapeutic options can be further subdivided into breast-conserving surgery, mastectomy, and surgical interventions of the axilla (sentinel lymph node biopsy and axillary lymph node dissection). Ultimately, each treatment decision should consider multiple factors such as the patient’s age and history, the TNM stage of the tumor, the presence of metastases, as well as its distinctive histological characteristics and tumor biology [6,7,8,9]. 

An important paradigm-changer in terms of treatment has been made in systemic therapy. Nowadays chemotherapy is more and more frequently performed neoadjuvant, prior to the operation, with significant advantages compared with adjuvant regimens [10,11]. On the one hand, the administration of neoadjuvant chemotherapeutic agents allows for testing the tumor response directly in vivo, which confers significant oncologic benefit to the patient, but also invariably enables dealing with research questions regarding the underlying tumor biology [12,13]. On the other hand, it leads to increased chances of breast-conserving surgery instead of ablative procedures [14,15,16,17]. In comparison to adjuvant chemotherapy, there is no disadvantage for patients in terms of overall survival and recurrence-free survival [16]. Within this context, accurate measurement of all residual tumor areas post-chemotherapeutically plays a crucial role for planning and selecting surgical approaches. As pathological complete response (pCR, id est, lack of invasive tumor areas and/or intraductal disease as well as the absence of tumorous lymph node affection) was shown to be associated with both an improved long-term prognosis and a reduced risk of local recurrence; its occurrence after neoadjuvant chemotherapy is a key prognostic factor [17,18,19,20]. Established clinical and imaging methods used for determination of the neoadjuvant tumor size as well as the assessment of the patient’s response to neoadjuvant chemotherapy include clinical examination via manual palpation, mammography, and breast ultrasonography [13]. The aim of this study is to compare the aforementioned methods and final postoperative pathological findings after neoadjuvant chemotherapy, intending to document the diagnostic accuracy of each method and, at best, to determine its ability to predict complete remission and the tumor status of resection margins, or at least to identify patients in whom surgical re-resection could be avoided. Within this validation-based research mission, the clinical significance of our study results contributes to existing and highly clinically relevant knowledge aimed at optimized and evidence-based treatment decisions in oncology.

## 2. Materials and Methods

Retrospective data from the hospital’s internal documentation system on 223 patients with breast carcinoma were initially reviewed and screened for eligibility. A priori defined exclusion criteria were: multifocality of breast carcinomas and extensive co-existence of ductal carcinoma in situ (DCIS). In total, clinico-pathological data from 184 patients with 193 tumors who were diagnosed with breast carcinoma and received neoadjuvant chemotherapy at the Department of Gynecology, Obstetrics and Reproductive Medicine at Saarland University in the ten years assessed (2008–2017) were included in this study (see Supplementary Table S1 for detailed clinical data of all tumor cases included). Our study protocol was planned in line with all requirements of the Ethics Committee of Saarland (study identification number 214/17); all data were stored, analyzed, and processed according to the “EU General Data Protection Regulation” (2018) as well as the Declaration of Helsinki [21].

For initial diagnosis, all patients with a lesion suspicious of breast carcinoma underwent punch biopsy with subsequent histomorphological tissue analysis; therefore, the diagnosis of breast carcinoma of all patients enrolled in this study was histopathologically assessed (light microscopy), including a determination of the intrinsic subtypes viz. “Luminal A”, “Luminal B”, “HER2”, “Basal like”, by means of additional immunohistochemical analyses [22]. All tumors were marked (clipped) immediately after diagnosis, a standard procedure marking the initial area of first tumor occurrence pre-therapeutically which allows for safe tumor area identification and removal in case of proven pCR. All patients included in the study were pre-staged according to national guidelines [23]; the cancer staging of all patients enrolled was M0 according to the TNM Classification of Malignant Tumors (UICC/AJCC staging system) [24]. All patients underwent regular monitoring during neoadjuvant chemotherapy and were consistently evaluated according to “RECIST” criteria (response evaluation criteria in solid tumors). Patients further underwent a thorough clinical/radiographic examination including manual palpation, breast ultrasonography, and mammography of the affected side in 2 planes approximately 3 weeks after completion of neoadjuvant chemotherapy [25]. The regimen of neoadjuvant chemotherapy was administered either as outlined in the guideline or as determined in ongoing clinical trials, mostly as a combination of anthracyclines and taxanes as well as eventually carboplatin in case of triple-negative breast carcinoma (TNBC). Each breast ultrasonography examination was performed by a gynecologist (either a resident with special training in breast ultrasonography or a board-certified specialist) and mammography was assessed by both residents as well as specialists in radiology (applying the four-eyes principle). Additional MRI examinations were not generally required for all patients but rather performed solely in individual cases. In order to achieve the optimal result for the individual clinical course of each patient, consultation with the surgeon was conducted prior to planned surgery, which was in general performed approximately 3–4 weeks after the last administration of chemotherapy. 

Preoperative clinical and imaging examinations of the breast were all performed in conformity with in-house standards and according to good clinical practice. The postoperative radiological control was carried out within one week (7 days) after completion of the neoadjuvant therapy. Standard craniocaudal and mediolateral oblique measurements were obtained during examination to determine the maximum tumor diameter. Signs of potential malignancy detectable on mammography scans were defined as dense, nodular lesions exhibiting poorly delineated or spiculated tumor margins as well as clusters of calcification or distortion/loss of physiological tissue architecture, whereas signs of potential malignancy on breast ultrasonography were defined as distinct hypoechoic lesions harboring poorly delineated tumor margins as well as acoustic shadowing or prominent vascularization [26]. Routine histomorphological tissue assessment was carried out after gross processing of the formalin-fixed tumor specimens; subsequent macroscopically identified tumor areas and their surroundings were embedded in paraffine wax and initially evaluated on hematoxylin and eosin (H&E)-stained slides. Whenever necessary, further diagnostics such as additional immunohistochemical staining or fluorescence in situ hybridization were conducted within the routine pathological workflow. In case of total remission to neoadjuvant chemotherapy, pathological complete response (pCR) was diagnosed. In the case of residual tumor areas, the extent of the tumor bed was measured by means of light microscopy, and the largest diameter served as our ground truth which we included in the statistical analysis [26].

When selecting a cut off value for the comparison of measurements, the permissible range of accuracy was set within ±1 cm. All categorical variables were descriptively analyzed by absolute and relative frequency counts. All continuous variables were described by the number, mean, and standard deviation of non-missing observations. In addition, the median, minimal, and maximal values and the 1st and 3rd quartile were calculated. The correlation between continuous variables was assessed using the Pearson and Spearman correlation coefficients. The agreement between classifications was assessed by the kappa statistic. Following Landis and Koch, a kappa < 0 was interpreted as no, 0–0.20 as slight, 0.21–0.40 as fair, 0.41–0.60 as moderate, 0.61–0.80 as substantial, and 0.81–1 as almost perfect agreement [27]. ROC curves were calculated and compared using DeLong’s test. A comparison-wise significance level of 5% was used, as appropriate for exploratory analyses. As far as missing variables are concerned, all patients with evaluable data were analyzed.

## 3. Results

The median age of the 184 patients enrolled in this study was 53 years. Of all 193 tumors, 163 (84.46%) could be assessed mammographically prior to as well as after neoadjuvant chemotherapy. However, a total number of 30 tumors (15.54%) could not be clearly delineated due to the lack of a representative, circumscribed lesion. Sonographically, a measurement of the tumor was feasible in 192 cases (99.48%) post-chemotherapy and in all cases prior to chemotherapy. Breast ultrasonography was performed by a specialist in gynecology in 119 cases and by a resident with special training in breast sonography in 74 cases. In total, 177 (91.71%) palpation findings were documented after completion of chemotherapy. 

The primary sonographically measured mean tumor size was 2.4 cm, whereas the mammographically measured mean tumor size was 2.6 cm. In terms of breast density, almost as many patients with low breast density (ACR 1 + 2) were enrolled as with dense breast density (ACR 3 + 4) (50.26% vs. 47.67%). Regarding the respective TNM classification, lymph node involvement was present in 46.11% of cases, a G3 tumor was present in 54.92% of our patients, and 67 tumors were triple-negative (34.72%); see Table 1 for details and tumor characteristics. 

Overall, mammographic measurements most accurately matched with the pathologically determined tumor size (±1 cm) in over 80% of cases. Breast ultrasonography measurements corresponded with the accurate pathological tumor measurement (±1 cm) in 73.96% of cases, with the latter overestimating maximal tumor extent by more than 10% (26.04% vs. 16.56%) in comparison to mammography (Table 2). In 107 (55.44%) of the 193 tumors, pCR (ypT0) was demonstrated after neoadjuvant chemotherapy. A total of 41 of the 107 patients were Her2neu positive and 39 were TNBC, viz. 41/65 of Her2neu positive patients (63.08%) and 58.21% of TNBC patients achieved a pCR. Moreover, 74.77% (80/107) of patients who achieved a pCR had aggressive tumors. Of patients who had G3 tumors, 62.26% (66/106) achieved a pCR. A pCR after neoadjuvant chemotherapy was correctly classified by means of manual palpation in 104 tumors (97.20%), by means of breast sonography in 61 tumors (57%), and by means of mammography in 87 tumors (81.31%). Table 3 compares the individual values in millimeters (mm) measured during manual palpation, breast ultrasonography, and mammography, with the values received at final histopathological examination after neoadjuvant therapy; the weighted kappa values of 0.47 for mammography and 0.52 for breast ultrasonography can be considered as moderate. For more in-depth information, the initial sonographic and mammographic BIRADS classification, the ypT stadium, the grade of neoadjuvant histological regression, the pCR rates based on molecular subtypes, and the initial cT-stage are displayed in Supplementary Tables S2 and S3 and Figure S1. In addition, we examined whether the expertise of the examiner (experienced and well-trained resident vs. specialist with years of experience in mammary sonography) mattered in terms of accuracy and precision in performing the sonographic examination post-chemotherapy and found no significant difference.

**Figure 1:**
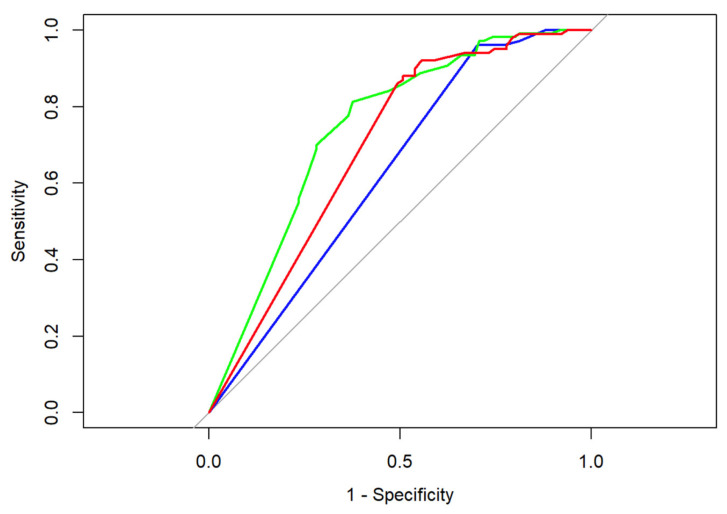
ROC curves for breast ultrasonography (green), mammography (red), and manual palpation (blue); corresponding AUC values are 0.75 (breast ultrasonography), 0.70 (mammography), and 0.63 (manual palpation). Baseline performance that corresponds to random chance classification is represented in grey.

Looking at our data (Table 4) we determined that both radiographical and sonographical tumor assessments, as well as measurements of manual palpation, led partially to both false negatives (rate: number of false negatives divided by number of all patients with histomorphologically identified malignant cells) and false positives (rate: number of false positives divided by number of all patients with pCR). In the case of false positive results, a measurable lesion could be determined in the imaging assessment or during clinical examination, which did not prove to be a malignant tumor in the histomorphological analysis, whereas in case of false negative results no distinct clinical lesion was present, albeit invasive tumor cells could be determined by means of light microscopy. Measurements conducted via breast ultrasonography led to the highest rate of false positives (46.02%); bimanual palpation led the highest rate of false negatives (69.12%) [28]. Comparing the ROC curves as well as the empiric AUC values of the respective imaging modality/examination method, breast ultrasonography outperforms manual palpation in the prediction of neoadjuvant tumor size as determined during final histopathological examination (DeLong’s test: Z = −2.3095, *p*-value = 0.02092); for more details, see Figure 1. 

## 4. Discussion

Breast ultrasonography allows the clinical assessment and measurement of almost any focal lesion. However, due to improved resolution of ultrasound devices nowadays, structural tissue changes in the original tumor area can still be visualized after neoadjuvant chemotherapy, even if the pathologist no longer determines any vital tumor remnants postoperatively. This phenomenon potentially explains the high false positive rate of 46.02% using breast ultrasonography for tumor assessment after chemotherapy. The expertise of the respective examiner (experienced resident or a bord-certified specialist in gynecology) did not show a significant difference within our work. Therefore, it can be concluded that breast ultrasonography as a method per se is not able to predict pCR with confidence, regardless of the experience of the examiner. Our data suggest that a resident trained in breast ultrasonography can perform the sonographic examination as well as a long-time specialist.

In our work, the estimated neoadjuvant tumor size (±1 cm) by means of sonography was in line with the pathological result in 73.96% of cases. Compared with the other published work in the literature, our numbers are slightly better (Keune et al. succeeded in 59.6% of cases [26]) or similar [28]. In the work published by Chagpar et al., the estimated neoadjuvant tumor size (±1 cm) agreed with the pathological result in 75% of cases; the authors enrolled 189 patients in total [28]. Another work published by Vriens et al. showed that breast ultrasonography was able to correctly estimate tumor size in 63% of cases. In this work, a comparison between MRI and ultrasound regarding their capabilities in predicting the correct pathological tumor size after neoadjuvant chemotherapy was studied; breast sonography performed significantly better with 63% compared to MRI with an accuracy of only 54% [29].

The mammographically reported tumor size (±1 cm) corresponded with the pathological result in 83.44% of our cases, a result significantly better than the results of comparable, previously published studies. For instance, the mammographically determined tumor size (±1 cm) matched the neoadjuvant pathological tumor size in only 70% of cases in the work of Chagpar et al. and in only 31.7% of cases in the work of Keune et al. [26,28]. 

To assess the agreement of clinically measured tumor size with the actual tumor size measured by the pathologist after neoadjuvant chemotherapy, this study demonstrated weighted kappa values for mammography of 0.4700 and for breast sonography of 0.5155. Our results are consistent with the literature: Chagpar et al. determined kappa values of 0.35 for mammography and 0.30 for breast sonography in their work [28]. Other publications such as that of Shin et al. follow suit with kappa values of 0.44 for mammography and 0.5 for breast ultrasonography; in the work by Shin et al., only MRI was able to achieve kappa values of 0.82 [30]. By contrast, the study published by Keune et al. reported a kappa of 0.4 for mammography and a kappa 0.45 for breast ultrasonography [26]. Overall, our results are in the moderate range; kappa values below 0.4 represent a poor agreement. 

Regarding diagnostic accuracy, the positive predictive value (PPV) for identifying residual tumor areas was 77.22% for breast ultrasonography and 74.36% for mammography, which is in line with the results published by Croshaw et al. in 2011. In their work, the authors determined a PPV > 75% for all methods [31]. Our analysis showed a FNR (false negative rate) for breast ultrasound of 22.78% and a NPV of 53.98%. Our results are consistent with those of Schaefgen et al., who demonstrated a NPV of 51.0% and a FNR of 24.3% for breast ultrasonography [32]. In contrast, our FNR values for mammography are slightly worse and the NPV slightly better compared to the aforementioned publication, namely a NPV of 65.22% and a FNR of 50.0% within our patient cohort vs. a NPV of 48.1% and a FNR of 30.3% reported by Schaefgen et al. [32]. Potentially, the high FNR for mammography of our analysis could be related to the low number of enrolled patients.

Since the commonly employed imaging modalities did not prove sufficient for a precise detection of pCR, alternative diagnostic methods such as minimal invasive biopsy were put to test. Theoretically, re-biopsy after neoadjuvant chemotherapy could increase the accuracy of prediction, although there are plausible concordant pitfalls, namely whether the area of interest is correctly hit. Generally, a biopsy can be regarded as a random sample. If the result is negative, it cannot be assumed with certainty that the tumor has regressed in the entire area. Under chemotherapeutic treatment, tumors can disintegrate quite diffusely, like a “Swiss cheese”. Therefore, surgery with the removal of the total tumor area is often the safest option for the patient. A study by Heil et al. showed in a cohort of 50 patients that a representative ultrasound-guided vacuum biopsy (VAB) yielded reliable histopathological findings regarding postoperative pathological determination of pCR. However, this was solely possible in 38 of the 50 patients; the NPV was 94.4% (95% CI 87.1–100.0) and the FNR was 4.8% (95% CI 0.0–11.6). About 24% of VABs remained unrepresentative due to poor sonographic visualization of the target lesion [33]. It remains to be noted that the small number of cases, only 50 patients, is a limitation of the above-mentioned work. Additional work presented at the Breast Cancer Symposium 2019 in San Antonio showed relatively high false negative rates with pCR detection for both core-needle biopsies (30–50%) and vacuum-assisted breast biopsies (17–19%):

The multicenter, prospective RESPONDER study (NCT02948764), in which a sonographically guided vacuum biopsy was performed in 79% of cases and a stereotactic vacuum biopsy in 21% of cases, proved that in 10% of cases the specimens have been classified as unrepresentative by pathological tissue assessment. Furthermore, a false negative rate (FNR) of 17.8% (95% CI: 12.8–23.7) was determined [34,35]. The second study involving 166 patients to determine residual disease via biopsy achieved a FNR of 18.7% (95% CI: 9.8–26.8). Each biopsy was conducted in either a sonographically (*n* = 129) or stereotactically (*n* = 37) controlled manner, and representative biopsies were obtained from the tumor area in 159 patients (95.78%) [36]. The third study (NRG-BR005 study), presented by Basik et al. including 105 patients, also failed to demonstrate reliable pCR prediction via biopsy. A negative predictive value (NPV) ≥ 90% was not achieved; only 50% of patients with residual tumor were detected after neoadjuvant chemotherapy (NCT) [37]. The fourth phase II study presented, MICRA, which involved a total of 167 patients from the Netherlands, also did not demonstrate reliable pCR detection by means of biopsy. In 89 patients (53%), pCR was correctly diagnosed solely based on biopsies taken after neoadjuvant systemic therapy (false positive rate, 0%); however, tumor residuals were not detected based on these biopsies in 29/78 patients (FNR, 37%). Additionally, MRI did not show reliable pCR detection in patients in this study [38]. In summary, surgery after neoadjuvant chemotherapy remains the gold standard for determining pCR to date.

### Limitations

One limitation of our study is the analysis of an entirely retrospective data set. Since all data were collected during routine clinical workflow, a certain intra- and interobserver variability on part of both radiologists and gynecologists must be assumed, although no significant bias could be demonstrated within our study. However, the involvement of different people as well as different specialties are a given and consistent clinical fact in Germany, which necessarily results from the competence and the associated legal framework of the respective (sub-)specialty. Therefore, our study design reflects the actual clinical situation in a realistic manner, aiming for maximized generalizability. Furthermore, the small number of just under 200 patients should be mentioned as another limitation of our study. 

## 5. Conclusions

In the present study, we operationalized both radiological methods, specifically mammography and breast ultrasound, as well as a clinical examination method, manual palpation. We compared their ability to determine the size of breast cancer after neoadjuvant chemotherapy with postsurgical histopathological measurements of the same tumor; the latter served as our ground truth. The conducted study shows that breast ultrasonography is significantly superior in comparison to manual palpation after completed neoadjuvant chemotherapy to predict a pCR; therefore, special focus should be placed on this particular modality during clinical follow up. However, none of the presented methods put to test were able to predict either a pCR or the potential effect of tumor margins with a high certainty.

## Figures and Tables

**Table 1:** Baseline and distinct tumor characteristics (tumor entity, TNM stage, hormone receptor as well as her2/neu status, mammographic breast density) of a total of 193 tumors (186 patients enrolled). ACR: American College of Radiology.

Clinical Tumor Characteristics	Total Number of Tumors *N* = 193 (%)
Tumor type	
NST (no special type)	175 (90.67)
Other than NST	18 (9.33)
Tumor stage	
T1	79 (40.93)
T2	91 (47.15)
T3	9 (4.66)
T4	14 (7.25)
Nodal status	
N0	103 (53.37)
N positive	89 (46.11)
Nx	1 (0.52)
Grading	
G1+2	83 (43.01)
G3	106 (54.92)
Gx	4 (2.07)
Estrogen receptor	
Positive	101 (52.33)
Negative	92 (47.67)
Progesterone receptor	
Positive	71 (36.79)
Negative	122 (63.21)
Her2/neu receptor	
Positive	65 (33.68)
Negative	128 (66.32)
Triple-negative	67 (34.72)
Breast density	
ACR 1 + 2	97 (50.26)
ACR 3 + 4	92 (47.67)
ACR unknown	4 (2.07)

**Table 2:** Performance of mammography, breast ultrasound, and manual palpation in assessing neoadjuvant tumor size compared with postoperative histopathological measurements. Accurate estimation as well as the over- and underestimates for each method are presented as individual characteristics (columns).

Imaging Modality	Accurate ±1 cm *n* (%)	Overestimation >1 cm *n* (%)	Underestimation >1 cm *n* (%)	Total Number of Tumors Assessed
Palpation	144 (81.36)	9 (5.09)	24 (13.56)	*n* = 177 (91.71)
Sonography	142 (73.96)	50 (26.04)	0 (0)	*n* = 192 (99.48)
Mammogram	136 (83.44)	27 (16.56)	0 (0)	*n* = 163 (84.46)

**Table 3:** Comparison between clinical measurements of mammography, breast ultrasonography, and manual palpation in assessing neoadjuvant tumor size with the gold standard of postsurgical histomorphological measurements. * To determine an agreement between classifications, the kappa statistic following Landis and Koch was employed; kappa < 0 was interpreted as no, 0–0.20 as slight, 0.21–0.40 as fair, 0.41–0.60 as moderate, 0.61–0.80 as substantial, and 0.81–1 as almost perfect agreement. ** Numbers within the diagonal display a close agreement between clinical measurements of tumor size pre-surgically as well as afterwards by means of light microscopy.

Clinical Measurements (mm)	Pathologic Measurements (mm)					
Physical Examination	0	0–10	11–20	21–30	>30	Weighted Kappa *
0	104 **	27	18	2	0	0.4273
1–10	0	1 **	2	2	0	
11–20	4	1	5 **	2	0	
21–30	1	0	0	2 **	0	
>30	0	0	0	3	3 **	
**Sonography**						
0	61 **	12	5	1	0	0.5155
1–10	38	13 **	11	1	0	
11–20	12	5	11 **	5	0	
21–30	1	0	2	6 **	2	
>30	1	0	0	1	4 **	
**Mammogram**						
0	87 **	21	9	0	0	0.4700
1–10	10	4 **	3	2	0	
11–20	5	4	4 **	1	0	
21–30	0	0	3	4 **	0	
>30	1	0	1	3	1 **	

**Table 4:** Diagnostic accuracy measures for each method put to test. Within our analysis, breast ultrasonography showed the highest rate of false positives (46.02%) whereas bimanual palpation showed the highest rate of false negatives (69.12%). FPR: false positive rate, FNR: false negative rate, NPV: negative predictive value, PPV: positive predictive value.

Clinical Measurement	FPR (%)	FNR (%)	Sensitivity (%)	Specificity (%)	NPV (%)	PPV (%)
Physical examination	4.59% (5/109)	69.12% (47/68)	95.41% (104/109)	30.89% (21/68)	80.77% (21/26)	68.87% (104/151)
Ultrasonography	46.02% (52/113)	22.78% (18/79)	53.98% (61/113)	77.22% (61/79)	53.98% (61/113)	77.22% (61/79)
Mammogram	15.53% (16/103)	50.00% (30/60)	84.47% (87/103)	50.00% (30/60)	65.22% (30/46)	74.36% (87/117)

## Data Availability

The data presented in this study are available upon request from the corresponding author.

## Supplementary Materials

The following supporting information can be downloaded at: https://www.mdpi.com/article/10.3390/diagnostics13172811/s1. Figure S1: Alluvial plots demonstrated the change of cT to ypT-stage after chemotherapy. Stages correspond to the TNM classification; Table S1: Chemotherapeutic treatment for the tumors included in the study. Data of all tumor cases included; Table S2: Summary statistics of preoperative ultrasound and mammography classification along with ypT stadium, the grade of neoadjuvant histological regression, and the molecular subtype of each tumor case determined by means of immunohistochemistry; Table S3: Distribution of pathological complete response (pCR) and ypT-stage among different molecular types of breast cancers included in the study.
