# Supplementary material for: Accuracy of Breast Ultrasonography and Mammography in Comparison with Postoperative Histopathology in Breast Cancer Patients after Neoadjuvant Chemotherapy
Source: Diagnostics (Basel). 2023 Aug 30;13(17):2811. doi: 10.3390/diagnostics13172811 (PMC10486727; doi:10.3390/diagnostics13172811)

Supplementary Material

Title: Accuracy of breast ultrasonography and mammography in comparison with postoperative histopathology in breast cancer patients after neoadjuvant chemotherapy

Supplementary Table S1. Chemotherapeutic treatment for the tumors included in the study. Data of all tumor cases included.

**Chemotherapeutic medication**	**Frequency (%)**
Epi/nabPacli/Cyclo (similar to ETC)	2 (1.0%)
Epi/Cyclo	3 (1.6%)
Epi/Cyclo /Doc/Beva	3 (1.6%)
Epi/Cyclo/Pacli/Carbo	11 (5.7%)
Epi/Cyclo/Doc	42 (21.8%)
Epi/Cyclo/Doc/Pacli	2 (1.0%)
Epi/Cyclo/nabPacli	8 (4.1%)
Epi/Cyclo/Pacli	30 (15.5%)
Epi/Cyclo/Pacli/Beva	1 (0.5%)
ETC	20 (10.4%)
LipDoxo/Pacli/Carbo	3 (1.6%)
Pacli	1 (0.5%)
Pacli/Beva	1 (0.5%)
Pacli/Carbo	1 (0.5%)
Doxo/Pacli/Carbo	2 (1.0%)
TAC	11 (5.7%)
**Combinations with anti-Her2neu therapies**	
Doxo/Pacli/Carbo/Trastuzumab, Pertuzumab	1 (0.5%)
ETC/Trastuzumab, Pertuzumab	5 (2.6%)
Epi/Cyclo/Doc/Trastuzumab, Pertuzumab	2 (1.0%)
Epi/Cyclo/Doc/Lapatinib	1 (0.5%)
Epi/Cyclo/Doc/Trastuzumab	8 (4.1%)
Epi/Cyclo/nabPacli/Trastuzumab, Pertuzumab	1 (0.5%)
Epi/Cyclo/Pacli/Trastuzumab, Pertuzumab	20 (10.4%)
Epi/Cyclo/Pacli/Trastuzumab	6 (3.1%)
ETC/Trastuzumab	2 (1.0%)
Pacli/Trastuzumab, Pertuzumab	2 (1.0%)
LipDoxo/Pacli/Trastuzumab, Pertuzumab	1 (0.5%)
TCH	1 (0.5%)
TCHP	2 (1.0%)

Beva: Bevacizumab, Carbo: Carboplatin, Cyclo: Cyclophosphamide, Doc: Docetaxel, Dox: Doxorubicin, Epi: Epirubicin, ETC: Epirubicin, Paclitaxel, Cyclophosphamid (Intense Dose-Dense Sequential Chemotherapy), LipDoxo: Liposomales Doxorubicin, nabPacli: nab-Paclitaxel, Pacli: Paclitaxel, TAC: Docetaxel, Adriamycin, Cyclophosphamide, TCH: Paclitaxel, Carboplatin, Trastuzumab, TCHP: Paclitaxel, Carboplatin, Trastuzumab, Pertuzumab

Supplementary Table S2. Summary statistics of preoperative ultrasound and mammography classification according to Breast Imaging Reporting & Data System (BI-RADS®) along with ypT stadium, the grade of neoadjuvant histological regression as well as the molecular subtype of each tumor case determined by means of immunohistochemistry. ypT = pathological T value (tumor diameter) of the postsurgical TNM stadium.

Variable	Frequency (%)
BIRADS Sonography	
2	1 (0.5%)
3	1 (0.5%)
4[1]	41 (21.2%)
4a	3 (1.6%)
4b	3 (1.6%)
4c	11 (5.7%)
5	85 (44%)
6	47 (24.4%)
Missing	1 (0.5%)
BIRAS Mammography	
1	2 (1.0%)
2	9 (4.7%)
3	6 (3.1%)
4[1]	30 (15.5%)
4a	5 (2.6%)
4b	3 (1.6%)
4c	6 (3.1%)
5	94 (48.7%)
6	33 (17.1%)
Missing	5 (2.6%)
ypT-Stadium	
0	107 (55.4%)
Tis	6 (3.1%)
1	60 (31.1%)
2	15 (7.8%)
3	2 (1.0%)
4	3 (1.6%)
Regression grade (nach Sinn)	
0	3 (1.6%)
1	43 (22.3%)
2	30 (15.5%)
3	6 (3.1%)
4	107 (55.4%)
Missing/unclear	4 (2.1%)
Biological subtype	
Luminal A	19 (9.8%)
Luminal B	42 (21.8%)
Her2-like	65 (33.7%)
Triple negative	67 (34.7%)

[1]provided only when further classification in 4a-c was not possible.

Supplementary Table S3 . Distribution of pathologic complete response (pCR) and ypT-Stage among different molecular types of breast cancers included in the study. Signficant differences based on the chi-squared test can be seen ($p < 0.001$),

ypT	Luminal A	Luminal B	Her2-like	Triple negative
0	6 (31.6%)	16 (38.1%)	46 (70.8%)	39 (58.2%)
Tis	0	1 (2.4%)	5 (7.7%)	0
≥ 1	13 (68.4%)	25 (59.5%)	14 (21.5%)	28 (41.8%)

Supplementary Figure S1. Alluvial plot demonstrated the change of cT to ypT-Stage after chemotherapy. Stages correspond to the TNM classification.

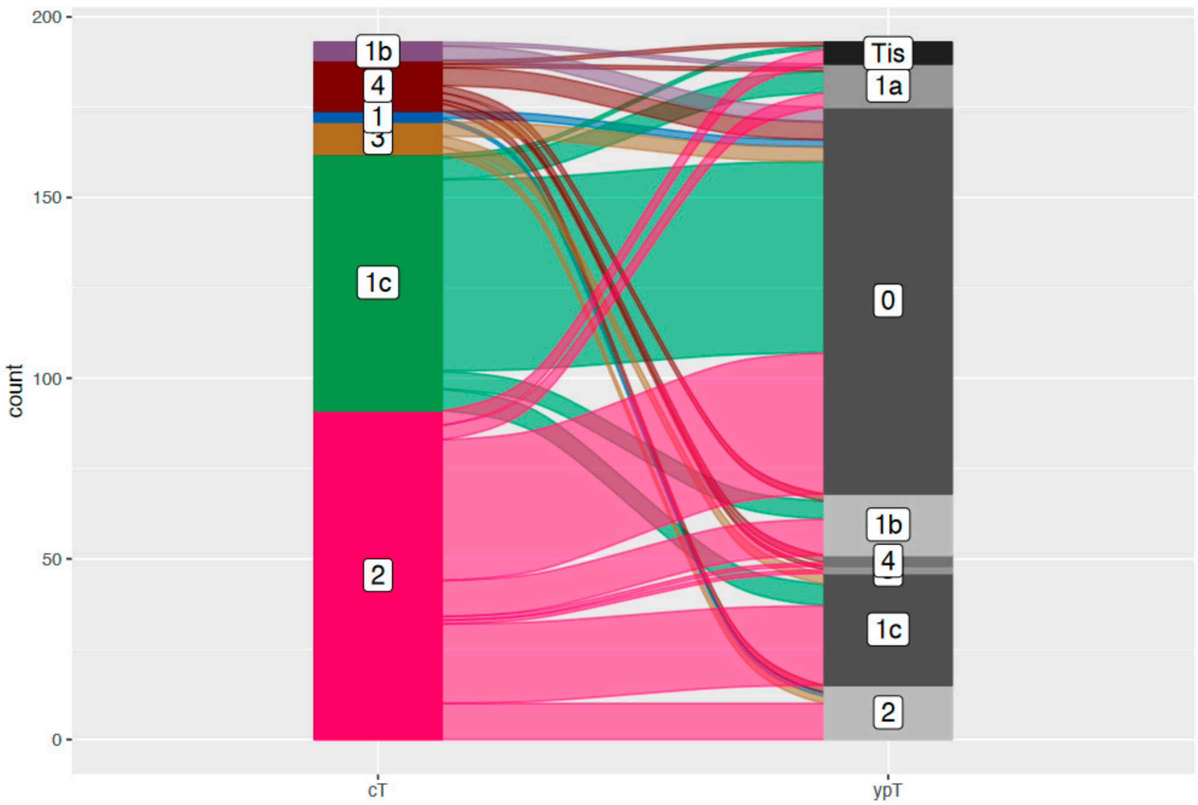

Supplement: Supplementary file 1 [file diagnostics-13-02811-s001.zip › diagnostics-2486405-supplementary.pdf]
